# Characterization of bacterial community associated with phytoplankton bloom in a eutrophic lake in South Norway using 16S rRNA gene amplicon sequence analysis

**DOI:** 10.1371/journal.pone.0173408

**Published:** 2017-03-10

**Authors:** Niranjan Nitin Parulekar, Pandurang Kolekar, Andrew Jenkins, Synne Kleiven, Hans Utkilen, Anette Johansen, Sangeeta Sawant, Urmila Kulkarni-Kale, Mohan Kale, Mona Sæbø

**Affiliations:** 1 Department of Natural Sciences and Environmental Health, Faculty of Technology, Natural Sciences and Maritime Sciences, University College of Southeast Norway, Bø i Telemark, Norway; 2 Bioinformatics Centre, Savitribai Phule Pune University (formerly University of Pune), Pune, India; 3 Department of Statistics, Savitribai Phule Pune University (formerly University of Pune), Pune, India; University of Wisconsin Milwaukee, UNITED STATES

## Abstract

Interactions between different phytoplankton taxa and heterotrophic bacterial communities within aquatic environments can differentially support growth of various heterotrophic bacterial species. In this study, phytoplankton diversity was studied using traditional microscopic techniques and the bacterial communities associated with phytoplankton bloom were studied using High Throughput Sequencing (HTS) analysis of 16S rRNA gene amplicons from the V1-V3 and V3-V4 hypervariable regions. Samples were collected from Lake Akersvannet, a eutrophic lake in South Norway, during the growth season from June to August 2013. Microscopic examination revealed that the phytoplankton community was mostly represented by *Cyanobacteria* and the dinoflagellate *Ceratium hirundinella*. The HTS results revealed that *Proteobacteria (Alpha*, *Beta*, *and Gamma)*, *Bacteriodetes*, *Cyanobacteria*, *Actinobacteria* and *Verrucomicrobia* dominated the bacterial community, with varying relative abundances throughout the sampling season. Species level identification of *Cyanobacteria* showed a mixed population of *Aphanizomenon flos-aquae*, *Microcystis aeruginosa* and *Woronichinia naegeliana*. A significant proportion of the microbial community was composed of unclassified taxa which might represent locally adapted freshwater bacterial groups. Comparison of cyanobacterial species composition from HTS and microscopy revealed quantitative discrepancies, indicating a need for cross validation of results. To our knowledge, this is the first study that uses HTS methods for studying the bacterial community associated with phytoplankton blooms in a Norwegian lake. The study demonstrates the value of considering results from multiple methods when studying bacterial communities.

## Introduction

In recent years, reports of cyanobacterial blooms in freshwater ecosystems attributed to eutrophication and global warming have increased [1–6]. *Cyanobacteria* rapidly form massive water blooms under favorable conditions, causing economic, ecological and health problems [7–9]. Many bloom-forming species produce toxins and the types of toxin produced are dependent on the species composition within the bloom [10–12]. *Cyanobacteria* coexist and interact with heterotrophic bacterial groups commonly found in freshwater ecosystems [13, 14]. They may produce various dissolved organic compounds and fix atmospheric nitrogen, supplying heterotrophic bacteria with substrates for growth [15–17]. Such compounds can differentially support growth of heterotrophic bacteria species [18]. Therefore, it is likely that the composition of cyanobacterial blooms will structure the associated heterotrophic bacterial populations [14].

Studies on phytoplankton cultures demonstrated a larger difference in bacterial community structure between diatom and *cyanobacteria* as compared to different cyanobacterial cultures. This is dependent on phytoplankton growth phase and the habitat of the associated bacterial community (free-living within bloom vs attached to the phytoplankton cell surface) [18]. In freshwater ecosystems, environmental conditions, including water temperature, residence time and mixing, light intensity and quality, nutrient concentrations and grazing pressure affect the development of cyanobacterial population [19].

Blooms in temperate regions, occur mostly during late summer and early autumn [6, 20] and are dominated by *Microcystis*, *Aphanizomenon*, *Anabaena* together with the invasive species e.g. *Cylindrospermopsis raciborskii* [21, 22]. Freshwater lakes are numerous in the Nordic countries: Norway (65,000 lakes), Sweden (95,700 lakes) and Finland (187,888 lakes) [23]. Almost 40% of Norwegian water bodies are at risk from human influences such as acidification and eutrophication; climate change is likely to exacerbate these problems in the future (www.environment.no, Freshwater 2015). Reports have shown that increase in water temperature in major European lakes, coupled with decreased duration of ice cover, has caused changes in the life cycle of phytoplankton and promoted the invasion of toxic species from warmer regions [3, 24].

Previous, culture-based, studies in Finland and Sweden have identified freshwater bacterial groups *Bacteriodetes*, *Proteobacteria*, *Firmicutes*, *Planctomycetes*, *Verrucomicrobia*, *Acidobacteria*, *Chloroflexi*, and *Thermomicrobia* in association with cyanobacteria, along with potentially pathogenic bacterial groups such as *Pseudomonas*, *Aeromonas* and *Vibrio* [13, 14].

Culture-dependent methods have shortcomings, as up to 95% of the microorganisms may be uncultivable [25–27]. High throughput sequencing, in contrast to culture dependent studies, can detect a large majority of microbial taxa present. This helps generate a deeper understanding when comparing bacterial communities [18, 28]. In this study we have used HTS to assess the bacterial community associated with phytoplankton during the blooming season in the eutrophic Norwegian lake Akersvannet by targeting two hypervariable regions, V1-V3 and V3-V4, of the 16S rRNA gene. To the best of our knowledge, this is the first study of the bacterial community in a freshwater ecosystem in Norway using HTS 16S rRNA amplicon sequencing.

## Materials and methods

### Study site and sampling

**Fig 1:**
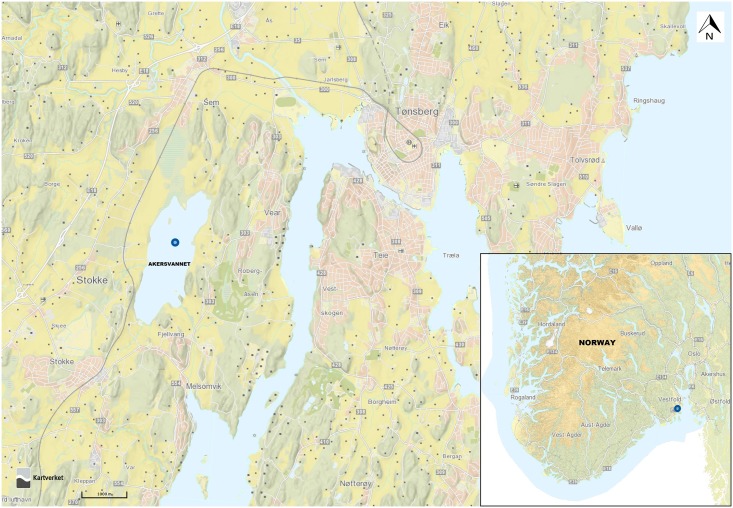
Map showing Lake Akersvannet. The sampling point is indicated by a blue circle [source; Mapping Authority (Norwegian Geographical Survey), www.norgeskart.no]. The insert in the right-hand corner shows the position of the lake on the map of southern Norway.

Lake Akersvannet is situated in Stokke Municipality, Vestfold County (South Norway). The lake has a mean depth of 6 m, maximum depth of 13 m and a total area of 2.4 km^2^ (Fig 1). It is an important wetland habitat for birds during the spring and autumn migration. During summer and fall cyanobacterial blooms occur frequently [29]. The lake is ice-covered during the winter. The lake is surrounded by agricultural land and is used for recreational activities including water sports and fishing. The first documented bloom at this lake was identified as *Aphanizomenon flos-aquae* (NIVA 1959). Lowering of the water level due to canalization during the year 1968 led to a shift in species composition. By the year 1994, the phytoplankton community was dominated by species of *Anabaena* and *Aphanizomenon* and the dinoflagellate *Ceratium hirundinella* [30].

This study was conducted in close cooperation with Vestfold County administration and the group of land owners surrounding the lake. In accordance with “Section IV” of the “Preservation regulations for Lake Akersvannet nature reserve” (www.lovdata.no) and “Section 3.3” of the “Management plan for Lake Akersvannet” *(*www.fylkesmannen.no), specific permission for taking water samples for research and educational purposes is not needed. Water samples were collected monthly from June to August 2013. Samples were taken at the sampling point indicated in Fig 1 (59.25° N 10.33° E). Separate one liter water samples were collected at depths of 0 m, 2 m and 4 m respectively. Net samples (25 μm pore size) for microscopic analysis were collected at the same time and location. Samples were immediately transferred to a cooled chamber and stored at 4°C.

### Chemical analysis

The chemical analysis of water quality parameters was performed in accordance with Norwegian standards (NS): pH (NS 4720), oxygen (NS 5813), total nitrogen (TN) (NS 4743), total phosphorus (TP) (NS 4725, 1984) and chlorophyll a (NS 4766 (1/1983)). Ammonia (NH_4_^+^) and nitrate (NO_3_^-^) were analyzed on Dionex ICS 1100 ion chromatograph (Thermo Fisher Scientific, California, USA) according to the manufacturer’s instructions.

### Toxin analyses

Enzyme-Linked Immunosorbent Assay technique (ELISA) with Microcystin ADDA ELISA Kit (product No. 520011) from Abraxis (Abraxis LLC, Warminster PA) was used for the microcystin analyses. All samples were freeze-thawed twice to lyse the cells and release the microcystins into the water. An AccuReader (Metertech, Taiwan) model 965 was used to read the absorbance at 450 nm.

### Qualitative and quantitative analyses of phytoplankton

Net samples were fixed with Lugol solution and examined under 100 and 400 x magnification for qualitative determination of the phytoplankton community (S1 Table). Phytoplankton enumeration was performed by Utermöhl inverted microscope technique using a 10ml sedimentation chamber. Species identification was performed according to Tikkanen and Willén [31] (S2 Table).

### DNA extraction

For each sampling date, three 1L samples, collected at depths of 0 m, 2 m and 4 m were available. Each sample was filtered through four to five 1.2 μm pore size, 47 mm diameter GF/C filters (Whatman, Norway). Each set of filters was cut into fragments in mm-sized pieces with a disposable sterile scalpel blade and incubated in XS buffer for 120 min and subjected to DNA extraction using the Xanthogenate-SDS (XS) method [32]. DNA pellets were washed twice with 70% ethanol, air-dried and suspended in 50μl TE buffer (10 mM Tris-HCL, 1 mM EDTA pH 8). The DNA extract from each set of filters were pooled to prepare one DNA pool per sampling depth. DNA pools from each sampling depth (0, 2 and 4 m) were then combined, giving an integrated sample. This integrated sample was then split into two equal volumes (replicates), and each replicate was used as a template for amplification and sequencing of V1-V3 and V3-V4 target regions of the 16S rRNA gene.

### PCR amplification and paired-end illumina sequencing

PCR amplification and sequencing was performed at Biomedicum Functional Genomics Unit, University of Helsinki, Finland. Each DNA replicate was used as template for amplifying two hypervariable regions of the 16S rRNA gene, using primers pairs V1-V3-27F (AGAGTTTGATCMTGGCTCAG), V1-V3-519R (GWATTACCGCGGCKGCTG) [33], V3-V4-341F (CCTACGGGNGGCWGCAG) and V3-V4-805R (GACTACHVGGGTATCTAATCC) [34]. Illumina adapter overhang sequences were attached to the 5’ end of each primer. PCR amplification and sequencing was performed according to Illumina’s 16S Metagenomic Sequencing Library Preparation guide (Part # 15044223 Rev. B). After PCR amplification the libraries were quantified using a Qubit Fluorometer (dsDNA HS) and the size of each library was estimated using the Agilent 2100 Bioanalyzer (DNA HS). The PCR libraries were normalized, pooled and then sequenced using the Illumina MiSeq System. Sequencing was performed using MiSeq 2 × 300 bp paired-end strategy and MiSeq Reagent Kit V3. For each replicate, as PCR amplicons from two different 16S hypervariable regions were pooled together and sequenced, no further barcoding was required [35].

### Data analysis

The sequence reads were analyzed using UPARSE v8.0.1623 [36], which allows accurate identification of Operational Taxonomic Units (OTU`s) (http://drive5.com/usearch/manual/uparse_pipeline.html). The raw paired-end sequence reads were merged using the command *usearch -fastq_mergepairs* with maximum expected error threshold set at 1.0. The merged reads were filtered on the basis of sequence quality *using fastq_filter* program, setting maximum expected error threshold to 0.5 and minimum sequence length to 250 bp. After quality filtering, the reads were aligned against the 16S rRNA sequences of the RDP database using the *Aligner* tool [37] in the RDP pipeline available at Ribosomal Database Project (RDP Release 11, Update 4), and endpoints were identified in order to allow their sorting into V1-V3 and V3-V4 reads. For both classes of reads, dereplication was performed to collapse identical reads into one single sequence. Following dereplication, abundance sorting was done and singletons were discarded. OTU`s were clustered at 3% divergence threshold and chimeras were eliminated using UCHIME [38]. Representative sequences for each OTU were selected and RDP classifier (16S rRNA training set 14) (S1 and S2 Texts) [39] was used to assign taxonomy. OTU’s assigned to *Archaea* and *Chloroplast* were filtered out and OTU tables were generated. Random subsampling of the OTU tables for both target regions (S3 and S4 Texts) was performed using script *single_rarefaction*.*py* in QIIME (Quantitative Insights Into Microbial Ecology package 1.9.1)[40]. Shared OTU’s between replicates were computed using script *shared_phylotypes*.*py* (S5 and S6 Texts). Diversity indices (Chao1, ACE and Shannon diversity) were calculated for the subsampled OTU tables using script *alpha_diversity*.*py*. The OTU’s were further classified using BLAST analysis [41] using nucleotide database (nt/nr) with uncultured/environmental sample sequences excluded. OTU’s were only assigned to a species, if the sequence similarity was ≥ 98% and no other species showed the same level of similarity. Where this was not achieved, the RDP taxonomic assignment was retained.

### Accession numbers

The raw 16S rRNA dataset from Lake Akersvannet sample are available at Sequence Read Archive (SRA) under study SRP095055.

## Results

The environmental conditions and phytoplankton biomass in Lake Akersvannet during the sampling season 2013 are shown in Table 1. TN ranged from 1.5 to 1.8 mg/l and TP varied between 90 and 148 μg/l. The highest water temperature (20.7°C) was recorded in August. The highest concentration of microcystin (0.5 μg/l) was detected in August. In June and July, the concentration of microcystin was below the detection limit of 0.15 μg/l.

**Table 1:** Environmental parameters and phytoplankton biomass in Lake Akersvannet during the sampling season 2013.

Environmental Parameters	June	July	August
Temp (°C) *	17.3 (± 0.3)	19.1 (± 0.6)	20.7 (± 2)
pH*	8.2 (± 0.1)	8.7 (± 0.3)	8.3 (± 0.5)
O_2_ (%)*	90 (± 2)	103 (± 9)	90 (± 15)
Total Nitrogen (mg/l) *	1.8 (± 164)	1.7 (± 30)	1.5 (± 300)
Total Phosphorus (μg/l) *	111(± 12)	90 (± 47)	148 (± 57)
Ammonia (μg/l) *	53 (± 92)	0	223 (± 76)
Nitrate (μg/l) *	1590 (± 310)	1530 (± 62)	710 (± 16)
Chlorophyll a (μg/l) **	9 (± 1)	30 (± 3)	52 (± 0)
Total phytoplankton biomass (mg/l) ***	0.4	1.4	16
Biomass of *Cyanobacteria* (mg/l) ***	0.1	1.2	0.1
*Cyanobacteria* (% of total phytoplankton)	24	77	1
Biomass of *Ceratium hirundinella* (mg/l) ***	0.04	0.05	15.4
*Ceratium hirundinella* (% of total phytoplankton)	11	3.5	96
Microcystin (μg/l) **	<0.15	<0.15	0.5 (± 0.8)

* mean values for depth 0-4m,

** mean values from photic zone,

*** mixed sample from photic zone.

Numbers in parentheses show standard deviation

### Phytoplankton community composition: Microscopic identification

Total phytoplankton biomass varied between 0.4 and 16 mg/l (Table 1) with mean biomass of 5.9 mg/l. Microscopic examination revealed variation in species composition of the phytoplankton community throughout the sampling season. *Cyanobacteria* represented 77% of total phytoplankton biomass in July, whereas the dinoflagellate *Ceratium hirundinella* dominated the community in August (96%). Species level morphological identification of *Cyanobacteria* showed the presence of *Aphanizomenon flos-aquae* (June-August), *Microcystis aeruginosa* (June-August), *Chroococcous turgidus* (June) and *Woronichinia naegeliana* (August). *Aphanizomenon* dominated the cyanobacterial community throughout the season. The proportion of *Microcystis* was increased in August (S2 Table).

### Bacterial community composition based on 16S rRNA amplicon sequencing

In total, 16,548 V1-V3 and 35,028 V3-V4 reads were obtained using RDP classifier (38). These were assigned to 255 and 502 bacterial OTU’s respectively.

Further analysis using BLAST allowed identification of 14 V1-V3 OTU’s at species level and three at genus level; similarly, 66 and nine V3-V4 OTU’s could be identified at species and genus level respectively (S3 Table). Six V1-V3 and 30 V3-V4 OTU’s, were highly similar (≥ 99%) to unclassified bacterial strain reported in other studies (S4 Table). Taxonomic classification using RDP classifier and BLAST were largely in agreement. There were two discrepancies at phylum level.

Bacterial richness and diversity indices (Alpha diversity) are shown in Table 2. Diversity was highest in July. Diversity was higher for the V3-V4 region than for V1-V3. Replicates gave comparable diversity estimates for each month, except August; August A had fewer OTU’s and lower diversity estimates. Comparison of phylotypes revealed that 76% of V1-V3 and 82% of V3-V4 OTU’s detected in August A were shared with August B (S5 and S6 Texts).

**Table 2:** Richness and Biodiversity indices for L. Akersvannet during the sampling season 2013.

Sampling month	Observed OTU’s (V1-V3)	Observed OTU’s (V3-V4)	Chao1 (V1-V3)	Chao1 (V3-V4)	ACE (V1-V3)	ACE (V3-V4)	Shannon diversity (V1-V3)	Shannon diversity (V3-V4)
June A	138	272	152	340	151	324	5.6	6.4
June B	141	272	157	333	161	329	5.5	6.5
July A	188	385	215	422	211	415	5.8	7.2
July B	179	389	201	403	202	407	5.8	7.3
August A	46	107	63	137	66	137	1.9	2.2
August B	86	187	161	246	158	242	2.7	3.5

**Fig 2:**
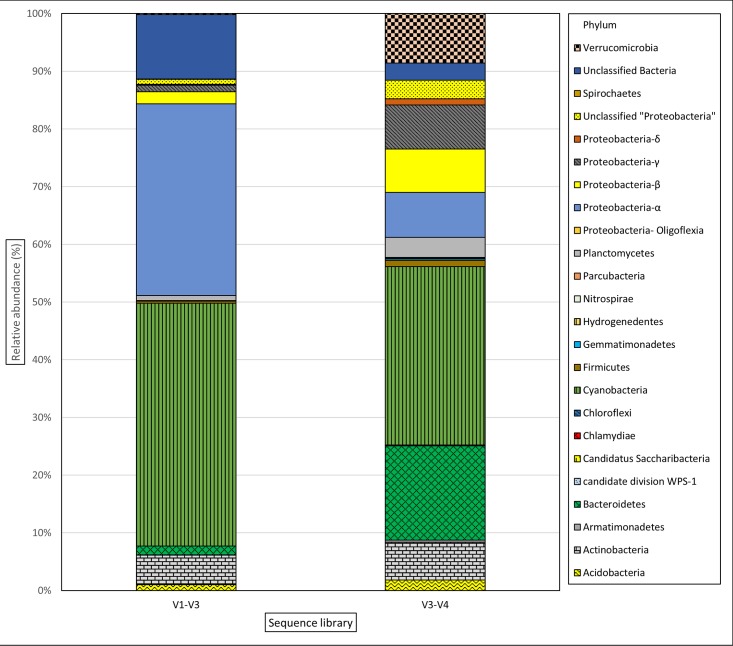
Phylum level distribution of reads for V1-V3 and V3-V4 hypervariable region.

After completion of taxonomic analysis, 255 V1-V3 OTU’s were assigned to 11 phyla and one group of “Unclassified Bacteria”, and 502 V3-V4 OTU’s were assigned to 17 phyla and one group of “Unclassified Bacteria”. A comparison of taxonomic distribution of reads between the two 16S rRNA target region libraries is shown in Tables 3 and 4. This comparison showed that 10 phyla were shared between both target regions, whereas respectively 1 and 7 phyla were exclusively identified in V1-V3 and V3-V4 sequence library. Phylum level distribution of reads for both the target regions is shown in Fig 2. The proportion of reads belonging to “Unclassified Bacteria” and *Proteobacteria-alpha* is higher in V1-V3 than V3-V4, whereas the proportion of *Verrucomicrobia* and *Bacteriodetes* is higher in V3-V4 sequence library. Overall proportions of unclassified OTUs were higher in the V1-V3 library (71%) than the V3-V4 (61%) (S5 Table).

**Table 3:** Comparison of taxonomic distribution of reads between V1-V3 and V3-V4 target regions.

Phylum	V1-V3 region			V3-V4 region		
	OTU count	Read count	Percent	OUT count	Read count	Percent
*Acidobacteria*	5	167	1.01%	20	640	1.83%
*Actinobacteria*	15	844	5.10%	31	2303	6.57%
*Armatimonadetes*	2	10	0.06%	5	113	0.32%
*Bacteroidetes*	23	259	1.57%	124	5732	16.36%
Candidate division WPS-1	0	0	0%	1	13	0.04%
*Candidatus* Saccharibacteria	0	0	0%	1	20	0.06%
*Chlamydiae*	0	0	0%	1	2	0.01%
*Chloroflexi*	0	0	0%	3	6	0.02%
*Cyanobacteria*	6	6958	42.05%	10	10834	30.93%
*Firmicutes*	1	65	0.39%	1	378	1.08%
*Gemmatimonadetes*	0	0	0%	3	96	0.27%
*Hydrogenedentes*	0	0	0%	1	37	0.11%
*Nitrospirae*	0	0	0%	1	12	0.03%
*Parcubacteria*	2	10	0.06%	6	28	0.08%
*Planctomycetes*	8	144	0.87%	24	1230	3.51%
Proteobacteria*	121	6201	37.47%	185	9534	27.22%
*Spirochaetes*	1	9	0.05%	0	0	0%
Unclassified Bacteria	69	1852	11.19%	47	1039	2.97%
*Verrucomicrobia*	2	29	0.18%	38	3011	8.60%
Total	255	16548	100%	502	35028	100%

* Please refer to Table 4 for class level breakup of phylum Proteobacteria.

**Table 4:** Comparison of class level taxonomic distribution of proteobacterial reads between V1-V3 and V3-V4 target regions.

Phylum *Proteobacteria*	V1-V3 region			V3-V4 region		
	OTU count	Read count	Percent	OTU count	Read count	Percent
*Proteobacteria- Oligoflexia*	0	0	0%	1	1	0.01%
*Proteobacteria-α*	82	5502	88.73%	66	2718	28.51%
*Proteobacteria-β*	14	346	5.58%	56	2644	27.73%
*Proteobacteria-γ*	8	185	2.98%	35	2665	27.95%
*Proteobacteria-δ*	2	30	0.48%	15	386	4.05%
Unclassified *"Proteobacteria"*	15	138	2.23%	12	1120	11.75%
Total	121	6201	100%	185	9534	100%

**Fig 3:**
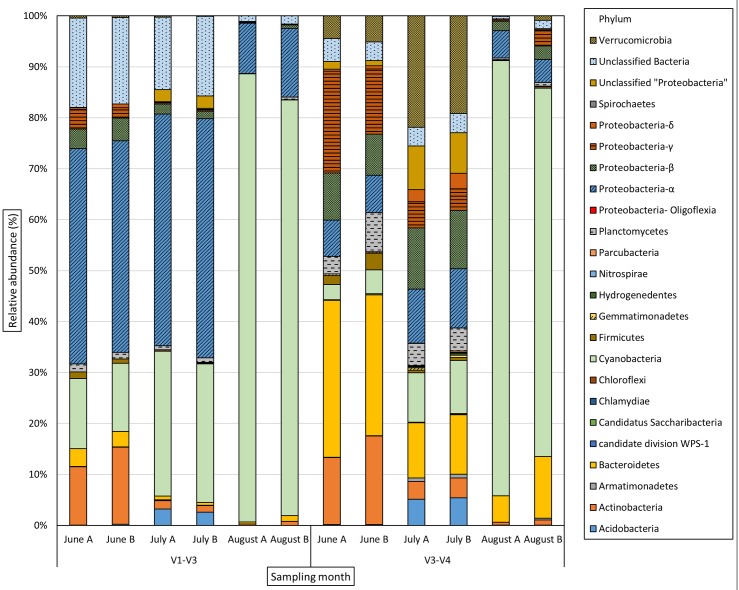
Phylum level distribution (%) of bacterial community in Lake Akersvannet from June to August 2013.

**Fig 4:**
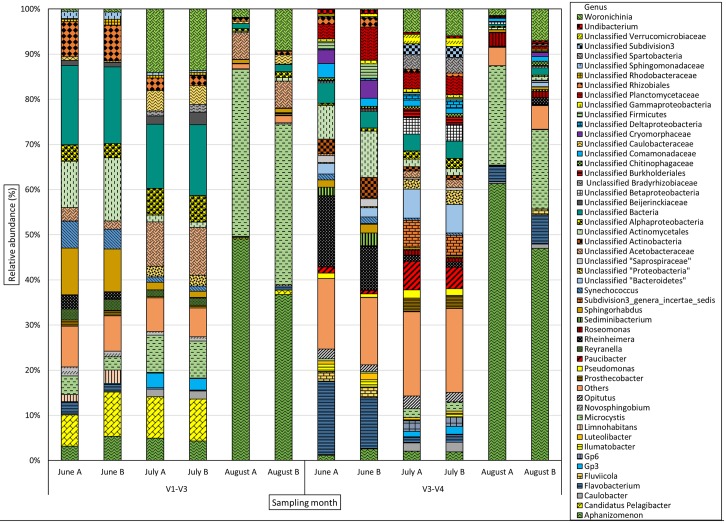
Genus level distribution (%) of bacterial community in Lake Akersvannet from June to August 2013. Genera with abundance <0.5% were combined and represented as “Others”.

**Fig 5:**
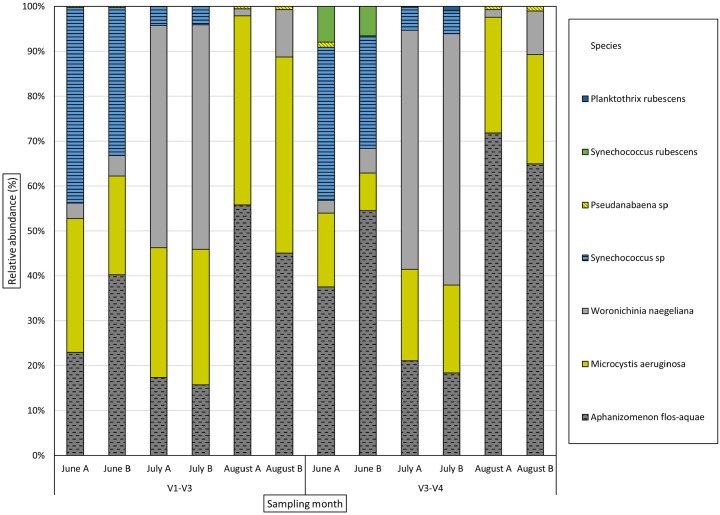
Species level distribution (%) of cyanobacterial community in Lake Akersvannet from June to August 2013.

The phylum and genus level bacterial community distribution is shown in Figs 3 and 4 respectively. The bacterial community was dominated by 5 phyla; namely *Actinobacteria*, *Proteobacteria*, *Cyanobacteria*, *Verrucomicrobia* and *Bacteriodetes* along with “Unclassified Bacteria” in varying proportions. *Cyanobacteria* were detected throughout the sampling period, but dominated both sequence libraries in August. Genera identified were *Aphanizomenon* (*GpI*), *Microcystis* (*GpXI*), *Synechococcus* (*GpIIa*), *Pseudanabaena* (*GpVI*), *Planktothrix* and *Woronichinia*. Species level distribution of total cyanobacterial reads is shown in Fig 5. The same dominant cyanobacterial genera and species were identified from both the 16S rRNA target regions (data in S3 Table).

### Comparison of microscopic and sequence-based cyanobacterial species distribution

The cyanobacterial species distribution derived from microscopic and sequence-based analysis is compared in Table 5. The same major species were found with both methods, but relative species abundance estimates differed.

**Table 5:** Comparison of microscopic and sequence-based cyanobacterial species distribution (L. Akersvannet 2013).

Identified	June			July			August		
	Mic	16S	16S	Mic	16S	16S	Mic	16S	16S
Cyanobacterial Species		(V1-V3)	(V3-V4)		(V1-V3)	(V3-V4)		(V1-V3)	(V3-V4)
*Aphanizomenon flos-aquae*	95.80%	31.5%	47.9%	99.80%	16.5%	19.7%	77.30%	50.62%	68.7%
*Microcystis aeruginosa*	2.10%	26%	11.5%	0.20%	29.5%	19.9%	14%	42.86%	25.1%
*Chroococcous turgidus*	2.10%	0%	0%	0%	0%	0%	0%	0%	0%
*Woronichinia naegeliana*	0%	4.0%	4.4%	0%	49.8	54.6%	8.70%	5.90%	5.4%
*Synechococcus* sp.	0%	38.3%	28.6%	0%	4.2	5.1%	0%	0%	0%
*Pseudanabaena* sp.	0%	0.3%	0.4%	0%	0%	0%	0%	0.62%	0.9%
Unclassified *Cyanobacteria*	0%	0%	0%	0%	0%	0.1%	0%	0%	0%
*Synechococcus rubescens*	0%	0%	7.1%	0%	0%	0.1%	0%	0%	0%
*Planktothrix rubescens*	0%	0%	0%	0%	0%	0.5%	0%	0%	0%
Total	100%	100%	100%	100%	100%	100%	100%	100%	100%

(Mic) Abundance (percentage of cyanobacteria) based on microscopic biomass estimation.

(16S) Abundance (percentage of cyanobacteria) based on 16S gene abundance data. Only cyanobacterial reads are included in the analysis. The values shown are the average of two replicates.

## Discussion

The mean phytoplankton biomass over the study period was 5.9 mg/l, making this lake hypereutrophic according to Brettum and Andersen [42]. Many factors regulate the composition of phytoplankton communities, but TN, TP and the ratio between TN and TP are considered particularly important [43, 44]. These parameters fluctuated moderately around means of TN 1.6 mg/l (SD ± 0.1 mg/l) and TP 116.3 μg/l (SD ± 23 μg/l) in this study. Although the low TN: TP ratios observed (10 to 18), were favorable for cyanobacterial blooms, the blooming organism was instead *C*. *hirundinella*, which also favors low TN: TP (i.e. < 7) as has been reported earlier in the literature [45–48]. It plausible that once a *Ceratium* bloom was established, it suppressed cyanobacterial bloom by competition for light and nutrients or by grazing [48, 49].

The increased proportions of *Cyanobacteria* observed in the 16S sequence library in August, could be due to the increase in water temperature (20.7°C) (Table 1). This temperature is optimal for growth of *Aph*. *flos-aquae* but suboptimal for *M*. *aeruginosa*. The lowest temperature at which *Aph*. *flos-aquae* can grow is reported to be 8°C [50]; optimum being 15 to 28°C [51], whereas *M*. *aeruginosa* can grow slowly at 15°C [52]; optimum being 27.5 to 32°C [51, 53]. *Aph*. *flos-aquae* has higher photosynthetic ability at lower temperatures as compared to *M*. *aeruginosa* [52]. In August, ammonium levels rose markedly and this was accompanied by an increase of *M*. *aeruginosa* (which favors ammonium as nitrogen source) [54, 55]. This would in turn explain the increase of microcystin in August.

### Comparison between the two target regions

Although less discriminatory than full length 16S rRNA sequencing, high throughput sequencing of shorter hypervariable regions (V1 to V9) using universal primers has become common practice in microbial community studies [35, 56]. Different hypervariable regions have been targeted in different studies with no one region being preferred over others [57, 58]. In our study we have utilized two of the most commonly used primer pairs, targeting the V1-V3 and V3-V4 region of the 16S rRNA gene respectively [59]. Of the ten phyla detected by both primer sets, five (*Cyanobacteria*, *Proteobacteria*, *Actinobacteria*, *Acidobacteria* and *Parcubacteria*) showed similar abundance profiles in both target sequence libraries, while the other five (*Bacteriodetes*, *Verrucomicrobia*, *Armatimonadetes*, *Firmicutes* and *Planctomycetes*) were markedly more abundant in the V3-V4 library. Eight phyla were detected by only one of the primer sets. This suggests that the primers used in this study selectively detect certain phyla and neither is truly universal [34]. For *cyanobacteria*, however, the results were highly concordant, suggesting that both primers sets have similar coverage for this phylum. Although the overall proportion of phylum *Proteobacteria* in both libraries was comparable, *Alphaproteobacteria* were higher in proportion in the V1-V3 library. This variation might be explained by higher coverage of V1-V3 primers for class *Alphaproteobacteria* as seen in studies conducted in other environment, but will require further experimentation to confirm these findings for freshwater environment [60]. The proportion of unclassified Bacteria and overall unclassified genera was greater in the V1-V3 library. Thus, use of different sets of universal primers can lead to different apparent community structures. During PCR amplification, target sequences that do not precisely match the universal primers will be either less efficiently amplified or lost completely [61] and use of particular sets of universal primers can lead to under- or over-estimation of taxa [35].

Comparison of community richness and diversity estimates showed greater diversity for V3-V4 than for V1-V3 libraries. Such discrepancies have been reported in other studies [35, 61]. Replicates gave comparable diversity estimates, except for samples August A and B. Even after random subsampling was performed, August A had fewer OTU’s than August B. Such discrepancies have been reported in other studies and attributed to errors in PCR amplification, library preparation or sequencing steps [61]. In our case as both target regions were equally depleted of OTUs in August A, the discrepancy is most probably due to differences in sequencing efficiency as V1-V3 and V3-V4 were sequenced together but amplified separately.

The bacterial community was dominated by five phyla; *Proteobacteria*, *Bacteriodetes*, *Cyanobacteria*, *Actinobacteria* and *Verrucomicrobia* in varying proportions throughout the study period. Genus level taxonomic identifications showed the presence of a great diversity of low abundance taxa. Previous studies refer to these low abundance taxa as “rare biosphere”. It has been suggested that members of the rare biosphere may play important roles in the bacterial community, despite their scarcity [18, 62]. In addition to the rare biosphere, many unclassified OTU’s were detected, suggesting that as-yet undescribed bacterial groups with unknown metabolic capabilities are an important part of the lake microbiota, a matter which warrants further investigation. The large proportion of unclassified bacterial taxa also suggests that taxa peculiar to the Norwegian freshwater environment may be absent from the cultured and sequenced species represented by commonly used 16S rRNA databases.

### Bacterial community distribution

*Bacteriodetes*, predominantly *Flavobacteriia* and *Sphingobacteriia* dominated the bacterial community throughout the sampling season. These are mostly chemoorganotrophs, that are well adapted to bloom conditions due to their ability to degrade complex biomolecules [18, 63, 64] such as algal-derived material [65] including cyanobacterial toxins [13]. *Flavobacteriia* and *Sphingobacteriia* have been found in high numbers during episodes of phytoplankton blooms [66], particularly cyanobacterial blooms in freshwater ecosystems [14, 67]. A study conducted in Sweden in 2007 reported multiple populations of *Flavobacteriia* in a bloom of cyanobacteria, without any seasonal succession and suggested that resource availability (organic matter) might be the driving force behind *Flavobacteriia* community structuring [64]. The genus *Flavobacterium*, is known to contain species that can lyse *Microcystis* cells [68] and degrade cyanobacterial hepatotoxins [13]. Member of this genus are also able to degrade complex organic molecules [64]. The dominant *Flavobacterium* taxon in June (OTU_11, V3-V4 library) showed high sequence similarity to a microcystin-degrading strain (*Flavobacterium* sp. AKB-2008 TE28) isolated in a previous study [13]. *Fluviicola*, another dominant genus of class *Flavobacteriia* detected in June and August is reported to contain species responsible for denitrification [69].

*Actinobacteria* have been found in various aquatic habitats and are considered to be ubiquitous members of the freshwater ecosystem, especially in the lake epilimnia [66, 70, 71]. In this study, the phylum was represented by *Actinomycetales* and *Acidimicrobiales*, two of the most abundant freshwater *Actinobacterial* orders [66, 72]. The ratio of allochthonous to autochthonous carbon sources, which may vary greatly during period of phytoplankton bloom, can be selective for different *Actinobacterial* species [73]. *Actinobacteria* have been previously associated with blooms of diatoms and *cyanobacteria* [14, 18, 71, 74, 75]. In this study, their abundance declined markedly from June to July while the cyanobacterial abundance increased. This might be due to water temperature increasing above the optimum for *Actinobacteria* [75] and/or competition from other, faster growing, bacterial phyla [76]. *Ilumatobacter*, one of the dominant *Actinobacteria* genera in June (V3-V4 library), are known to thrive in environments with elevated phosphorous levels [77], as found in L. Akersvannet.

In this study, *Verrucomicrobia* comprised 8.6% of the bacteria detected in the V3-V4 library. Although most studies report low abundance of *Verrucomicrobia*, much higher abundance was reported in a recent culture independent study [66, 78]. *Verrucomicrobia* abundance may have previously been underestimated due to poor cultivability and methodological biases [66, 78–80]. They are known degraders of algal polysaccharides and organic matter [81–83]. Hence their presence during a phytoplankton bloom is not unexpected [84]. A representative of *Subdivision3* (OTU_8, V3-V4 library), which dominated in July, showed high sequence similarity to a strain known to degrade laminarin, one of the most abundant algal polysaccharides in nature [83].

Most of the OTU’s belonging to *Actinobacteria* and *Verrucomicrobia*, detected in this study were highly similar to isolates previously identified in North American lakes [83, 85], which suggests that these strains are geographically widespread. Comparable reports, suggesting that unique capabilities of these groups allow them to compete successfully in lakes with diverse trophic states have been published [70, 86].

*Proteobacteria* represented a significant portion of the bacterial community, which is in agreement with other studies [74, 87, 88]. Many of the most prevalent and well-studied freshwater bacterial groups belong to *Betaproteobacteria* [66, 89]. *Paucibacter* (*Betaproteobacteria*) is represented by a single dominant OTU (OTU_6) which showed high sequence similarity to *Paucibacter toxinivorans*, a novel species isolated from lake sediment. *P*. *toxinivorans* can degrade the cyclic cyanobacterial hepatotoxins and mineralize organic phosphorous compounds [90]. However, the abundance of *P*. *toxinivorans* was higher in July (with toxin level below detection threshold) than in August (high toxin concentration detected), suggesting that the toxin presence is not critical for the growth of this species. *Limnohabitans*, one the betaproteobacterial genus identified in July (V1-V3 library), are opportunistic freshwater bacteria that prefer non-acidic environments [91–93]. Their ability to use low molecular weight algal exudates as sole organic carbon source (DOC) and rapid growth rate gives them a competitive advantage during phytoplankton bloom [76, 91, 94].

*Alphaproteobacteria* were the dominant proteobacterial group in this study. In contrast to *Betaproteobacteria*, *Alpha* and *Gammaproteobacteria* rarely dominate freshwater bacterial communities, except under conditions of elevated organic and inorganic inputs during phytoplankton blooms [13, 66, 76].

One of the *Gammaproteobacteria* OTU's found in this study was a *Rheinheimera* species. *Rheinheimera* are abundant in marine, freshwater and estuarine environments and are able to grow on and degrade organic matter [95–97]. Other *Gammaproteobacteria* were *Pseudomonas* and *Xanthomonadaceae*. These groups include opportunistic pathogens of humans, fish and plants [98, 99]. *Pseudomonas* and *Rheinheimera* are thought to enhance the growth of *Microcystis* by modulating phosphate exchange in the cyanobacterial mucilage capsule [13, 100].

*Sphingorhabdus* and *Novosphingobium* (*Alphaproteobacteria*) are physiologically diverse generalists capable of aerobic anoxygenic photosynthesis and degrading polycyclic aromatic compounds [75, 101–105]. *Candidatus* Pelagibacter, one of the dominant genera in the V1-V3 library, belongs to the *SAR11* lineage which is known to play important role in phosphate metabolism in aquatic ecosystems [106]. *Roseomonas*, a member of family *Acetobacteraceae*, has been previously been associated with cyanobacterial blooms and attaches to the cell surface of *M*. *aeruginosa* [14, 18]. Another *Alphaproteobacteria* species was *Caulobacter profundus* [107]. *Caulobacter* are known to be associated with cyanobacterial blooms and enhance the growth of *Microcystis* [13, 108]. The highest cyanotoxin concentration, in August, coincided with the greatest diversity of potentially cyanotoxin-degrading species like *Caulobacter*, *Novosphingobium*, *Sphingorhabdus (Alphaproteobacteria)*, *Paucibacter*, *Flavobacterium* (*Betaproteobacteria*) and *Pseudomonas* (*Gammaproteobacteria*). It has been suggested that cyanotoxins provide a competitive advantage to cyanotoxin-degraders by serving as nutritional supplement [109–111].

### Comparison of microscopic and sequence based results

The microscopic findings and 16S rRNA sequence analysis were in agreement in regard to the major species present. The quantitative results however were not in concordance. Similar findings have been reported previously [112, 113]. This discrepancy needs to be addressed, as it is commonly assumed that read counts are semi-quantitative and will provide meaningful relative abundance estimates [114]. A recent study has shown that *Cyanobacteria* were overrepresented in HTS data as compared to microscopic biovolume data. Thus, for the moment both methods are needed to obtain a picture of the cyanobacterial community structure [115].

The discrepancies observed indicate that there is significant bias in one or both of the methods used. Microscopy is a subjective method, dependent on the skill and experience of the microscopist and overestimation or underestimation of phytoplankton diversity may occur [112, 116, 117]. In addition, differential losses of cells may occur during fixation, depending on the species and cell size [113, 118, 119]. Smaller cells that do not sink fast enough within the counting chamber can be underrepresented or missed completely [115]. 16S amplicon read counts are subject to biases caused by cell lysis efficiency, DNA extraction efficiency, PCR amplification efficiency, varying 16S rRNA gene copy number, quality filtering criteria and the sequencing method used [14, 113, 114, 120–123]. Our results illustrate the importance of using multiple methods while characterizing freshwater microbiota. Additional experimentation is required to resolve the observed discrepancies.

## Conclusion

To our knowledge, this study is the first use of 16S rRNA targeted amplicon sequencing to characterize the bacterial communities associated with phytoplankton blooms in a eutrophic lake in Norway. Our study revealed that choice of 16S rRNA region can affect the apparent community structure and this needs to be taken into account in the planning and interpretation of such studies. Our results showed a highly complex and fluctuating microbial flora, mostly comprised of taxa previously found in association with phytoplankton blooms. A significant proportion of the community was represented by low abundance bacterial groups and unclassified taxa that might be performing hitherto unidentified ecological functions. Our study also showed that the resolving power and the quantitative results of microscopic and 16S rRNA targeted sequence analysis may differ, which needs further investigation.

The 16S rRNA results, provided a snapshot of the phytoplankton-associated bacterial community, at three individual time points. As phytoplankton communities are known to be highly dynamic, more frequent sampling will be needed to identify trends.

## Supporting information

S1 Table Taxonomic overview of phytoplankton in Akersvannet 2013 (Net samples). (DOCX)

S2 Table Total volume of phytoplankton in Akersvannet 2013 (Utermöhl method). (DOCX)

S3 Table Comparison of RDP and BLASTn taxon assignments: Species level (L. Akersvannet 2013). (DOCX)

S4 Table Comparison of RDP and BLASTn taxon assignment: Unclassified isolates (L. Akersvannet 2013). (DOCX)

S5 Table Distribution of unclassified OTUs between V1-V3 and V3-V4 target region. (DOCX)

S1 Text Representative OTU sequence for V1-V3 target region. (TXT)

S2 Text Representative OTU sequence for V3-V4 target region. (TXT)

S3 Text Subsampled OTU table for V1-V3 target region. (TXT)

S4 Text Subsampled OTU table for V3-V4 target region. (TXT)

S5 Text Shared phylotypes for V1-V3 target region. (TXT)

S6 Text Shared phylotypes for V3-V4 target region. (TXT)
