# Supplementary material for: Characterization of bacterial community associated with phytoplankton bloom in a eutrophic lake in South Norway using 16S rRNA gene amplicon sequence analysis
Source: PLoS One. 2017 Mar 10;12(3):e0173408. doi: 10.1371/journal.pone.0173408 (PMC5345797; doi:10.1371/journal.pone.0173408)
Supplement: S1 Table — (DOCX) [file pone.0173408.s001.docx]

**Table S1: Taxonomic overview of phytoplankton in Akersvannet 2013 (Net samples).**

| **Taxon** | **June** | **July** | **August** |
| --- | --- | --- | --- |
| ***Cyanophyceae*** |  |  |  |
| *Gompho*sp*haeria aponina* | X | X | X |
| *Woronichinia naegeliana* | X | X | X |
| *Microcystis aeruginosa* |  | X | X |
| *Chroococcus turgidus* | X | X |  |
| *Aphanizomenon flos-aquae* | X | X | X |
| *Anabaena* sp. |  |  |  |
|  |  |  |  |
| ***Cryptophyceae*** |  |  |  |
| *Cryptomonas* sp. |  | X | X |
| *Rhodomonas lacustris* | X |  |  |
|  |  |  |  |
| ***Dinophyceae*** |  |  |  |
| *Gymnodinium* sp. | X |  |  |
| *Ceratium hirundinella* | X | X | X |
| *Peridinium* sp. | X |  |  |
|  |  |  |  |
| ***Diatomophyceae*** |  |  |  |
| *Cyclotella* sp. |  |  | X |
| *Stephanodiscus* sp. |  |  |  |
| *Aulacoseira* sp. |  |  | X |
| *Aulacoseira granulata* |  | X | X |
| *Asterionella formosa* |  | X | X |
|  |  |  |  |
| ***Euglenophyceae*** |  |  |  |
| *Trachelomonas* sp. |  |  | X |
| *Trachelomonas volvocinopsis* |  |  |  |
| *Scourfieldia complanata* |  |  |  |
|  |  |  |  |
| ***Chlorophyceae*** |  |  |  |
| *Chlamydocapsa planctonica* | X | X | X |
| *Oocystis lacustris* |  |  | X |
| *Oocystis borgei* |  | X | X |
| *Oocystis* sp. |  |  |  |
| *Pediastrum duplex* | X | X |  |
| *Pediastrum boryanum* | X | X |  |
| *Coelastrum microporum* |  |  | X |
| *Coelastrum reticulatum* | X | X | X |
| *Kirchneriella obesa* |  | X |  |
| Sp*haerocystis schroeteri* |  |  | X |
|  |  |  |  |
| ***Conjugatophyceae*** |  |  |  |
| *Closterium* sp. |  | X |  |
| *Staurastrum tetracerum* |  |  |  |
| *Staurastrum anatinum* |  |  |  |
| *Staurastrum pingue* |  | X | X |
| *Staurastrum* sp. |  |  |  |
| **Total** | 12 | 17 | 18 |
