# Supplementary material for: Characterization of bacterial community associated with phytoplankton bloom in a eutrophic lake in South Norway using 16S rRNA gene amplicon sequence analysis
Source: PLoS One. 2017 Mar 10;12(3):e0173408. doi: 10.1371/journal.pone.0173408 (PMC5345797; doi:10.1371/journal.pone.0173408)
Supplement: S2 Table — (DOCX) [file pone.0173408.s002.docx]

**Table S2: Total volume of phytoplankton in Akersvannet 2013 (Utermöhl method).**

| ***Taxon*** | **June** | **July** | **August** |
| --- | --- | --- | --- |
|  | vol/l um^3^ | vol/l um^3^ | vol/l um^3^ |
| ***Dinophyceae*** |  |  |  |
| *Ceratium hirundinella* | 44268736 | 50032507 | 15389780442 |
|  |  |  |  |
| ***Spherical cells*** |  |  |  |
| *Spherical cells without flagella* | 86256587 | 172519477 | 136045034 |
| *Spherical cells with flagella* | 10654953 | 27525429 | 13461341 |
|  |  |  |  |
| ***Cyanophyceae*** |  |  |  |
| *Woronichinia naegeliana* | x | x | 11875772 |
| *Microcystis aeruginosa* | 2009155 | 2009155 | 18939809 |
| *Chroococcous turgidus* | 2009155 |  |  |
| *Aphanizomenon flos-aquae* | 93425726 | 1085449448 | 104877912 |
| *Sum* | 97444037 | 1085449464 | 135693493 |
|  |  |  |  |
| ***Cryptophyceae*** |  |  |  |
| *Cryptomonas* sp*.* | 2009155 | x | 160732432 |
| *Rhodomonas lacustris* | 151532420 | 29205005 | 16958540 |
| *Sum* | 153541576 | 29205005 | 177690972 |
|  |  |  |  |
| ***Diatomophyceae*** |  |  |  |
| *Stephanodiscus hantzschii* | 1808240 | 5424720 | x |
| *Aulacoseira* sp*.* |  | 4068540 | x |
| *Sum* | 1808240 | 9493259 |  |
|  |  |  |  |
| ***Chrysophyceae*** |  |  |  |
| *Mallomonas* sp*.* | x | x | 77151568 |
| *Synura* sp*.* | x | x | 1473381 |
| *Sum* |  |  | 78624948 |
|  |  |  |  |
| ***Euglenophyceae*** |  |  |  |
| *Chlamydomonas* sp*.* | 4018311 | 6429297 | x |
| *Scourfieldia complanata* | x | 2649109 | x |
| *Sum* | 4018311 | 15357017 | x |
|  |  |  |  |
| ***Chlorophyceae*** |  |  |  |
| *Pediastrum boryanum* | x | x | 57863676 |
| *Monoraphidium griffithi* | 602747 | x |  |
| *Scenedesmus Quadricauda* | 4018311 | x | 1797 |
| *Chlamydomonas* sp*.* | x | 6278611 | x |
| *Planktosphaeria gelatinosa* | x | 2712360 | x |
| *Keratococcus suecicus* | x | 602747 | x |
| *Coelastrum reticlutum* | x | 20590122 | x |
| *Sum* | 4621057 | 23905229 | 57865472 |
|  |  |  |  |
| **Diverse** | 5223804 | 2712360 | 11727038 |
|  |  |  |  |
| **Total** |  |  |  |
| um^3^/l | 407837301 | 1418208885 | 16000888741 |
| mm^3^/l | 0.408 | 1.418208885 | 16 |
|  |  |  |  |
