# Supplementary material for: Characterization of bacterial community associated with phytoplankton bloom in a eutrophic lake in South Norway using 16S rRNA gene amplicon sequence analysis
Source: PLoS One. 2017 Mar 10;12(3):e0173408. doi: 10.1371/journal.pone.0173408 (PMC5345797; doi:10.1371/journal.pone.0173408)
Supplement: S3 Table — (DOCX) [file pone.0173408.s003.docx]

**Table S3: Comparison of RDP and BLASTn taxon assignments: species level (L. Akersvannet 2013).**

| **OUT ID (V1-V3)** | **OUT ID (V3-V4)** | **RDP based genus level taxonomy** | **BLAST based species level taxonomy** | **Identity (%)** | **Accession entry (V1-V3 / V3-V4)** |
| --- | --- | --- | --- | --- | --- |
| OTU_1 | OTU_1 | *GpI* | *Aphanizomenon flos-aquae* | 99% | HE975012/ HE975012 |
| OTU_2 | OTU_3 | *GpXI* | *Microcystis aeruginosa* | 99% | CP012375/NR_074314 |
| OTU_4 | OTU_4 | Unclassified *Cyanobacteria* | *Woronichinia naegeliana* | 99% | AJ781043/AJ781043 |
| OTU_16 | OTU_37 | *GpIIa* | *Synechococcus* sp. | 98% | AF098373/AM259272 |
| OTU_80 | OTU_5 | *Flavobacterium* | *Flavobacterium aquatile* | 99% | NR_042495/NR_118482 |
| OTU_114 | OTU_368 | *GpIIa* | *Synechococcus* sp. | 99% | AM259272/AF448067 |
|  | OTU_414 | *Acidovorax* | *Acidovorax radicis* | 99% | /NR_117776 |
|  | OTU_115 | *Aeromonas* | *Aeromonas sobria* | 99% | /KX817242 |
|  | OTU_215 | Unclassified *Bradyrhizobiaceae* | *Afipia massiliensis* | 99% | /AB272322 |
|  | OTU_34 | *Algoriphagus* | *Algoriphagus* sp*. BAL317* | 99% | /KM586916 |
|  | OTU_79 | *Aquihabitans* | *Aquihabitans daechungensis* | 98% | /NR_132289 |
|  | OTU_211 | *Arcicella* | *Arcicella aurantiaca* | 99% | /NR_116735 |
|  | OTU_86 | Unclassified *Cytophagaceae* | *Arcicella* sp*.* | 99% | /JN408290 |
|  | OTU_227 | Unclassified *Xanthomonadaceae* | *Arenimonas subflava* | 98% | /NR_135888 |
|  | OTU_233 | Unclassified *Caulobacteraceae* | *Brevundimonas* sp. | 99% | /LN651199 |
|  | OTU_16 | Unclassified *"Saprospiraceae"* | Candidatus  *Aquirestis calciphila* | 99% | /AJ786342 |
|  | OTU_197 | Unclassified *Microbacteriaceae* | Candidatus  *Limnoluna rubra* | 99% | /NR_125497 |
|  | OTU_76 | Unclassified *Methylophilaceae* | Candidatus  *Methylopumilus planktonicus* | 99% | /LN827929 |
| OTU_3 |  | *Candidatus* Pelagibacter | Candidatus  *Pelagibacter* | 99% | JN941972/ |
| OTU_76 |  | Unclassified *Actinomycetales* | Candidatus  *Planktophila limnetica* | 99% | FJ428831/ |
|  | OTU_17 | *Caulobacter* | *Caulobacter profundus* | 98% | /KF360053 |
|  | OTU_141 | *Cellvibrio* | *Cellvibrio gandavensis* | 99% | /NR_025419 |
| OTU_270 |  | *Chryseobacterium* | *Chryseobacterium chaponense* | 99% | LN997906/ |
|  | OTU_306 | *Curvibacter* | *Curvibacter delicatus* | 98% | /NR_113696 |
|  | OTU_299 | *Deefgea* | *Deefgea chitinilytica* | 99% | /KC213956 |
| **OUT ID**  **(V1-V3)** | **OUT ID**  **(V3-V4)** | **RDP based genus level taxonomy** | **BLAST based species level taxonomy** | **Identity (%)** | **Accession entry (V1-V3 / V3-V4)** |
|  | OTU_286 | Unclassified *Rhizobiales* | *Devosia* sp*. HM-198* | 99% | /KP152608 |
|  | OTU_318 | *Elstera* | *Elstera litoralis* | 99% | /NR_116409 |
| OTU_134 |  | *Emticicia* | *Emticicia oligotrophica* | 98% | NR_074305/ |
|  | OTU_64 | *Emticicia* | *Emticicia sediminis* | 99% | /NR_136787 |
|  | OTU_274 | *Flavobacterium* | *Flavobacterium cheniae* | 98% | /NR_044198 |
|  | OTU_285 | *Flavobacterium* | *Flavobacterium cheonanense* | 98% | /NR_117493 |
|  | OTU_166 | *Flavobacterium* | *Flavobacterium columnare* | 98% | /NR_118582 |
|  | OTU_36 | *Flavobacterium* | *Flavobacterium pectinovorum* | 99% | /KY077148 |
|  | OTU_402 | *Flavobacterium* | *Flavobacterium squillarum* | 99% | /NR_109532 |
|  | OTU_124 | *Flavobacterium* | *Flavobacterium succinicans* | 99% | /JX657084 |
|  | OTU_21 | *Flavobacterium* | *Flavobacterium terrigena* | 99% | /JQ692099 |
|  | OTU_235 | *Flavobacterium* | *Flavobacterium yonginense* | 98% | /NR_108535 |
|  | OTU_104 | *Gemmatimonas* | *Gemmatimonas aurantiaca* | 98% | /NR_074708 |
|  | OTU_117 | Unclassified *Alphaproteobacteria* | *Hirschia* sp. | 99% | /KF056993 |
|  | OTU_314 | *Hyphomicrobium* | *Hyphomicrobium facile* | 98% | /NR_027610 |
|  | OTU_386 | *Ilumatobacter* | *Ilumatobacter fluminis* | 98% | /JQ899217 |
| OTU_207 |  | *Inhella* | *Inhella inkyongensis* | 99% | NR_043920/ |
| OTU_158 | OTU_107 | *Janthinobacterium* | *Janthinobacterium lividum* | 99% | EU275366/ KX12892 |
|  | OTU_188 | *Lacibacterium* | *Lacibacterium aquatile* | 98% | /KY114786 |
|  | OTU_295 | *Rhizobacter* | *Methylibium* sp. | 99% | /JQ977109 |
|  | OTU_121 | *Methylocystis* | *Methylocystis rosea* | 98% | /LT220849 |
|  | OTU_61 | *Methylophilus* | *Methylophilus methylotrophus* | 98% | /LC191544 |
|  | OTU_239 | *Mycobacterium* | *Mycobacterium mucogenicum* | 98% | /NR_042919 |
|  | OTU_394 | *Nitrosomonas* | *Nitrosomonas oligotropha* | 99% | /NR_104820 |
|  | OTU_480 | *Nitrospira* | *Nitrospira lenta* | 99% | /KF724505 |
|  | OTU_6 | Unclassified *"Proteobacteria"* | *Paucibacter toxinivorans* | 98% | /KU921562 |
|  | OTU_81 | *Pedobacter* | *Pedobacter boryungensis* | 98% | /KF528724 |
|  |  |  |  |  |  |
| **OUT ID (V1-V3)** | **OUT ID (V3-V4)** | **RDP based genus level taxonomy** | **BLAST based species level taxonomy** | **Identity (%)** | **Accession entry (V1-V3 / V3-V4)** |
|  | OTU_108 | Unclassified *Moraxellaceae* | *Perlucidibaca piscinae* | 99% | /NR_043919 |
|  | OTU_507 | Unclassified *Moraxellaceae* | *Perlucidibaca piscinae* | 99% | /KY114788 |
|  | OTU_324 | *Phenylobacterium* | *Phenylobacterium muchangponense* | 99% | /NR_117783 |
|  | OTU_374 | Unclassified *Cyanobacteria* | *Planktothrix rubescens* | 99% | /KY091689 |
|  | OTU_65 | *Polynucleobacter* | *Polynucleobacter asymbioticus* | 99% | /AB607317 |
|  | OTU_57 | *GpVI* | *Pseudanabaena mucicola* | 99% | /LC177664 |
| OTU_19 |  | Unclassified *Cyanobacteria* | *Pseudanabaena* sp. | 99% | AB936779/ |
|  | OTU_150 | *Pseudomonas* | *Pseudomonas migulae* | 99% | /LN995696 |
| OTU_12 |  | *Reyranella* | *Reyranella massiliensis* | 99% | HM048834/ |
|  | OTU_404 | *Reyranella* | *Reyranella soli* | 99% | /NR_109674 |
| OTU_122 |  | *Rheinheimera* | *Rheinheimera soli* | 99% | AM990696/ |
|  | OTU_114 | *Rhodobacter* | *Rhodobacter megalophilus* | 99% | /JQ692104 |
| OTU_93 |  | *Rhodoluna* | *Rhodoluna lacicola* | 98% | FJ545223/ |
|  | OTU_423 | *Sphingomonas* | *Sphingomonas starnbergensis* | 99% | /NR_118124 |
|  | OTU_32 | *Sphingorhabdus* | *Sphingopyxis rigui* | 99% | /KC157048 |
|  |  |  |  |  |  |
|  | OTU_204 | *Sphingorhabdus* | *Sphingopyxis wooponensis* | 99% | /NR_109148 |
| OTU_5 |  | *Sphingorhabdus* | *Sphingorhabdus rigui* | 99% | KC157048/ |
|  | OTU_246 | *Sulfuritalea* | *Sulfuritalea hydrogenivorans* | 98% | /NR_113147 |
|  | OTU_315 | *GpIIa* | *Synechococcus rubescens* | 99% | /NR_125481 |
|  | OTU_463 | *Terrimonas* | *Terrimonas lutea* | 98% | /NR_041250 |
|  | OTU_118 | *Undibacterium* | *Undibacterium seohonense* | 99% | /NR_125672 |
|  | OTU_438 | *Vogesella* | *Vogesella perlucida* | 99% | /KF911331 |
|  | OTU_485 | Unclassified *Rhizobiales* | *Woodsholea maritima* | 99% | /FM886859 |
|  |  |  |  |  |  |
