# Supplementary material for: Characterization of bacterial community associated with phytoplankton bloom in a eutrophic lake in South Norway using 16S rRNA gene amplicon sequence analysis
Source: PLoS One. 2017 Mar 10;12(3):e0173408. doi: 10.1371/journal.pone.0173408 (PMC5345797; doi:10.1371/journal.pone.0173408)
Supplement: S4 Table — (DOCX) [file pone.0173408.s004.docx]

**Table S4:** **Comparison of RDP and BLASTn taxon assignment: unclassified isolates (L. Akersvannet 2013).**

| **OUT ID**  **(V1-V3)** | **OUT ID**  **(V3-V4)** | **RDP based genus level taxonomy** | **Similarity to BLAST entry** | **Identity (%)** | **Accession entry**  **(V1-V3 / V3-V4)** |
| --- | --- | --- | --- | --- | --- |
| OTU_23 | OTU_18 | Unclassified *Actinomycetales* | *Actinobacterium* SCGC AAA208-D13 | 99% | JF488148/JF488148 |
| OTU_29 | OTU_7 | Unclassified Bacteria | *Firmicutes bacterium* | 99% | HQ663375/HQ663375 |
| OTU_30 | OTU_152 | Unclassified *Alphaproteobacteria* | *Hyphomonadaceae bacterium* | 99% | CP012156/CP012156 |
|  | OTU_60 | Unclassified *Acidobacteria_Gp3* | *Acidobacteria bacterium* SCGC AAA204-D14 | 99% | /JF488162 |
|  | OTU_12 | Unclassified *Actinomycetales* | *Actinobacterium* SCGC AAA041-M15 | 99% | /HQ663360 |
|  | OTU_73 | Unclassified *Actinomycetales* | *Actinobacterium* SCGC AAA041-O14 | 98% | /HQ663369 |
|  | OTU_262 | Unclassified *Actinomycetales* | *Actinobacterium* SCGC AAA043-K09 | 99% | /HQ663403 |
|  | OTU_48 | Unclassified *Actinobacteria* | *Actinobacterium* SCGC AAA043-M10 | 99% | /HQ663409 |
|  | OTU_80 | *Ilumatobacter* | *Actinobacterium* SCGC AAA043-M15 | 99% | /HQ663410 |
|  | OTU_476 | Unclassified *Actinobacteria* | *Actinobacterium* SCGC AAA044-D11 | 99% | /HQ663447 |
| OTU_10 |  | Unclassified *Actinomycetales* | *Actinobacterium* SCGC AAA208-N15 | 99% | JF488157/ |
|  | OTU_78 | Unclassified *Actinomycetales* | *Actinobacterium* SCGC AAA278-P09 | 99% | /HQ663606 |
|  | OTU_20 | Unclassified *Actinobacteria* | *Actinobacterium* SCGC AAA278-P23 | 99% | /HQ663610 |
|  | OTU_203 | Unclassified Bacteria | *Alpha proteobacterium* SCGC AAA160-M08 | 99% | /JF488597 |
|  | OTU_72 | *Candidatus* Pelagibacter | *Alpha- proteobacterium* SCGC AAA208-M13 | 99% | /JF488156 |
|  | OTU_13 | Unclassified *Rhizobiales* | *Bacterium* DR2A-55G25 | 99% | /AB127924 |
|  | OTU_209 | Unclassified *Chitinophagaceae* | *Bacterium* SCGC AAA018-G21 | 99% | /HQ290494 |
|  |  |  |  |  |  |
|  | OTU_161 | Unclassified *Cytophagales* | *Bacterium TG141 gene* | 99% | /AB308367 |
|  | OTU_10 | Unclassified Bacteria | *Bacteroidetes bacterium* SCGC AAA023-M10 | 99% | /HQ663022 |
|  | OTU_45 | Unclassified *Cryomorphaceae* | *Bacteroidetes bacterium* SCGC AAA027-D14 | 98% | /HQ663127 |
| **OUT ID**  **(V1-V3)** | **OUT ID**  **(V3-V4)** | **RDP based genus level taxonomy** | **Similarity to BLAST entry** | **Identity (%)** | **Accession entry**  **(V1-V3 / V3-V4)** |
|  | OTU_29 | *Sediminibacterium* | *Bacteroidetes bacterium* SCGC AAA206-C09 | 99% | /JF488130 |
| OTU_59 |  | *Caulobacter* | *Caulobacter sp.* AKB-2008-JO51 | 99% | AM989009/ |
|  | OTU_74 | Unclassified *Acidimicrobiales* | *Actinobacterium* SCGC AAA043-I09 | 99% | /HQ663397 |
|  | OTU_11 | *Flavobacterium* | *Flavobacterium sp.* AKB-2008-TE28 | 99% | /AM988932 |
| OTU_68 |  | Unclassified *Burkholderiales* | *Incertae sedis 5 bacterium* AKB-2008-JO111 | 99% | AM989123/ |
|  | OTU_22 | Unclassified *Planctomycetaceae* | *Planctomycetes bacterium* | 99% | /JF488132 |
|  | OTU_15 | *Rheinheimera* | *Rheinheimera sp.* AKB-2008-TE32 | 99% | /AM989306 |
|  | OTU_2 | *Rheinheimera* | *Rheinheimera sp.* IW-258 | 99% | /KF556702 |
|  | OTU_98 | Unclassified *Verrucomicrobiaceae* | *Verrucomicrobia bacterium* SCGC AAA027-I19 | 98% | /HQ663166 |
|  | OTU_31 | *Opitutus* | *Verrucomicrobia bacterium* SCGC AAA041-M18 | 99% | /HQ663361 |
|  | OTU_8 | *Subdivision3_genera_incertae_sedis* | *Verrucomicrobia bacterium* SCGC AAA204-G18 | 99% | /JF488114 |
|  | OTU_9 | Unclassified *Subdivision3* | *Verrucomicrobia bacterium* SCGC AAA204-K10 | 98% | /JF488119 |
|  | OTU_24 | Unclassified *Spartobacteria* | *Verrucomicrobia bacterium* SCGC AAA487-O09 | 99% | /HQ663696 |
