# Supplementary material for: Characterization of bacterial community associated with phytoplankton bloom in a eutrophic lake in South Norway using 16S rRNA gene amplicon sequence analysis
Source: PLoS One. 2017 Mar 10;12(3):e0173408. doi: 10.1371/journal.pone.0173408 (PMC5345797; doi:10.1371/journal.pone.0173408)
Supplement: S5 Table — (DOCX) [file pone.0173408.s005.docx]

**Table S5: Distribution of unclassified OTUs between V1-V3 and V3-V4 target region.**

| **Taxonomic assignment** | | **V1-V3 OTU count** | **V3-V4 OTU count** |
| --- | --- | --- | --- |
| Unclassified Bacteria | Unclassified Bacteria | 69 | 47 |
| *Acidobacteria* | Unclassified *Acidobacteria_Gp3* | 0 | 1 |
| *Actinobacteria* | Unclassified *Actinobacteria* | 1 | 4 |
|  | Unclassified *"Acidimicrobineae"* | 1 | 4 |
|  | Unclassified *Acidimicrobiaceae* | 1 | 0 |
|  | Unclassified *Actinobacteridae* | 0 | 1 |
|  | Unclassified *Actinomycetales* | 11 | 9 |
|  | Unclassified *Micrococcineae* | 0 | 3 |
|  | Unclassified *Microbacteriaceae* | 0 | 4 |
| *Armatimonadetes* | Unclassified *"Armatimonadetes"* | 1 | 1 |
| *Bacteroidetes* | Unclassified *"Bacteroidetes"* | 3 | 42 |
|  | Unclassified *Cytophagales* | 0 | 7 |
|  | Unclassified *Cytophagaceae* | 1 | 1 |
|  | Unclassified *"Flavobacteriales"* | 0 | 1 |
|  | Unclassified *Cryomorphaceae* | 3 | 7 |
|  | Unclassified *Flavobacteriaceae* | 0 | 1 |
|  | Unclassified *"Sphingobacteriales"* | 0 | 5 |
|  | Unclassified *"Saprospiraceae"* | 0 | 2 |
|  | Unclassified *Chitinophagaceae* | 0 | 14 |
|  | Unclassified *Sphingobacteriaceae* | 0 | 1 |
| *Chlamydiae* | Unclassified *Parachlamydiaceae* | 0 | 1 |
| *Chloroflexi* | Unclassified *Anaerolineaceae* | 0 | 1 |
|  | Unclassified *Caldilineaceae* | 0 | 1 |
| *Firmicutes* | Unclassified *Firmicutes* | 1 | 1 |
| *Planctomycetes* | Unclassified *"Planctomycetes"* | 3 | 0 |
|  | Unclassified *Phycisphaerae* | 1 | 0 |
| **Taxonomic assignment** | | **V1-V3 OTU count** | **V3-V4 OTU count** |
| *Planctomycetes* | Unclassified *Planctomycetaceae* | 0 | 16 |
| *Proteobacteria* | Unclassified *"Proteobacteria"* | 15 | 12 |
| *Proteobacteria-α* | Unclassified *Alphaproteobacteria* | 25 | 18 |
|  | Unclassified *Caulobacteraceae* | 3 | 4 |
|  | Unclassified *Rhizobiales* | 8 | 7 |
|  | Unclassified *Beijerinckiaceae* | 3 | 0 |
|  | Unclassified *Bradyrhizobiaceae* | 1 | 1 |
|  | Unclassified *Rhodobacteraceae* | 5 | 3 |
|  | Unclassified *Rhodospirillales* | 1 | 2 |
|  | Unclassified *Acetobacteraceae* | 11 | 6 |
|  | Unclassified *Rhodospirillaceae* | 1 | 0 |
|  | Unclassified *Rickettsiaceae* | 1 | 0 |
|  | Unclassified *Sphingomonadales* | 0 | 1 |
|  | Unclassified *Sphingomonadaceae* | 4 | 1 |
| *Proteobacteria-β* | Unclassified *Betaproteobacteria* | 2 | 17 |
|  | Unclassified *Burkholderiales* | 2 | 5 |
|  | Unclassified *Alcaligenaceae* | 0 | 1 |
|  | Unclassified *Comamonadaceae* | 0 | 4 |
|  | Unclassified *Oxalobacteraceae* | 1 | 1 |
|  | Unclassified *Methylophilaceae* | 0 | 1 |
|  | Unclassified *Neisseriaceae* | 0 | 2 |
|  | Unclassified *Rhodocyclaceae* | 0 | 3 |
| *Proteobacteria-δ* | Unclassified *Deltaproteobacteria* | 2 | 7 |
| *Proteobacteria-γ* | Unclassified *Bacteriovoracaceae* | 0 | 1 |
|  | Unclassified *Myxococcales* | 0 | 3 |

| **Taxonomic assignment** | | **V1-V3 OTU count** | **V3-V4 OTU count** |
| --- | --- | --- | --- |
| *Proteobacteria-γ* | Unclassified *Gammaproteobacteria* | 0 | 9 |
|  | Unclassified *Enterobacteriaceae* | 0 | 1 |
|  | Unclassified *Alteromonadaceae* | 0 | 1 |
|  | Unclassified *Chromatiales* | 0 | 1 |
|  | Unclassified *Moraxellaceae* | 0 | 3 |
|  | Unclassified *Sinobacteraceae* | 1 | 1 |
|  | Unclassified *Xanthomonadaceae* | 0 | 1 |
| *Spirochaetes* | Unclassified *Leptospiraceae* | 1 | 0 |
| *Verrucomicrobia* | Unclassified *"Verrucomicrobia"* | 1 | 2 |
|  | Unclassified *Opitutae* | 0 | 2 |
|  | Unclassified *Spartobacteria* | 1 | 4 |
|  | Unclassified *Verrucomicrobiaceae* | 0 | 8 |
| Total Unclassified OTU |  | 185 | 308 |
| Total OUT detected |  | 255 | 502 |
| Percent of Unclassified OTU |  | 72.55% | 61.35% |
